# Lithium-associated hyperparathyroidism: a systematic review of prevalence and surgical outcomes

**DOI:** 10.1007/s00423-026-04200-5

**Published:** 2026-08-17

**Authors:** Reza Alidousti Shahraki, Adrian D. Meehan, Göran Wallin, Kosmas Daskalakis

**Affiliations:** 1 Faculty of Medicine and Health, Department of Surgery, Örebro University, Örebro, Sweden; 2 Faculty of Medicine and Health, Department of Orthopaedic Surgery, Örebro University, Örebro, Sweden; 3 Second Department of Surgery, “Korgialenio-Benakio”, Red Cross General Hospital, Athens, Greece

**Keywords:** Lithium, Hyperparathyroidism, Hypercalcemia, Parathyroidectomy, Multiglandular disease, Calcium homeostasis, Bipolar disorder

## Abstract

**Purpose:**

Lithium remains one of the most effective maintenance treatments for bipolar disorder. However, disturbances in calcium homeostasis including hypercalcemia and lithium-associated hyperparathyroidism (LHPT) have increasingly been recognized as endocrine complications of chronic lithium therapy. The prevalence, pathophysiology, and optimal surgical management of LHPT remain incompletely defined. This systematic review aims to synthesize the available evidence on prevalence, pathophysiology, and surgical outcomes in LHPT.

**Methods:**

We conducted a systematic review according to PRISMA guidelines. Five databases (MEDLINE, Embase, Cochrane Library, Web of Science, and PsycINFO) were searched. Studies reporting hypercalcemia or hyperparathyroidism in adult patients receiving lithium therapy were eligible for inclusion. Surgical case series reporting parathyroidectomy in LHPT were analyzed separately.

**Results:**

A total of 5,483 records were identified, of which 125 studies met the inclusion criteria. Hypercalcemia occurred in 7.2% (423/5,853) and HPT was confirmed in 4.8% (156/3,239) of lithium-treated patients. Multiglandular disease was identified in 25–83% of surgical cases, substantially higher than the 15–20% observed in sporadic HPT. Bilateral neck exploration was performed in the majority of surgical series. Postoperative cure rates ranged from 58% to 100%, with recurrence rates of approximately 16% at 10 years.

**Conclusion:**

LHPT represents a clinically significant endocrine complication characterized by heterogeneous pathological patterns. The elevated prevalence of multiglandular disease has important implications for surgical planning. Given the high proportion of multiglandular involvement, surgeons should maintain a low threshold for bilateral neck exploration, particularly in patients with prolonged lithium exposure or intraoperative findings suggestive of multiple gland involvement.

**Supplementary Information:**

The online version contains supplementary material available at 10.1007/s00423-026-04200-5.

## Introduction

Lithium is one of the oldest known elements and occurs naturally on the earth as a monovalent cation [1, 2]. The Australian physician John Cade first demonstrated the therapeutic potential of lithium in the treatment of mania [3, 4]. Despite this early discovery, the introduction of lithium into routine psychiatric practice was initially hindered by several factors [5]. Subsequent work by Schou and colleagues confirmed the therapeutic efficacy of lithium, including one of the earliest randomized trials in psychopharmacology [3, 6]. Lithium is now widely regarded as a first-line maintenance therapy for bipolar disorder [7].

In clinical practice, lithium therapy is associated with a number of adverse effects affecting multiple organ systems. Commonly reported side effects include tremors, cognitive disturbances, thyroid dysfunction, and renal tubular abnormalities [8, 9]. Endocrine complications are particularly well recognized, with hypothyroidism occurring in a substantial proportion of lithium-treated patients [10]. Disturbances in calcium metabolism have also been described in association with lithium therapy. Hypercalcemia related to lithium treatment was first reported in the early 1970s [11]. Over time, lithium-associated hyperparathyroidism (LHPT) has been increasingly recognized as an important complication of chronic lithium therapy.

Before initiating lithium therapy, baseline investigations typically include evaluation of renal function, thyroid function, and electrolyte status, but only more recently has routine monitoring of serum calcium been incorporated into several clinical guidelines [12]. Parker et al. [13] have highlighted inconsistencies between guidelines regarding monitoring of lithium-associated metabolic complications.

Sporadic primary hyperparathyroidism (PHPT) is most commonly caused by single-gland disease and is often treated by focused minimally invasive parathyroidectomy or unilateral neck exploration [14]. In contrast, several studies suggest that LHPT may be characterized by a higher prevalence of multiglandular disease and parathyroid hyperplasia [15–31]. This observation has led some investigators to advocate bilateral neck exploration and subtotal parathyroidectomy in selected patients.

Given the increasing recognition of lithium-associated disturbances in calcium metabolism, a comprehensive synthesis of the available literature is warranted. The aim of the present study was therefore to identify studies reporting lithium-treated patients with disturbances in calcium homeostasis; to assess the prevalence of hypercalcemia and hyperparathyroidism; and to summarize current surgical strategies and outcomes in LHPT.

## Methods

### Study design and registration

This systematic review was conducted in accordance with the Preferred Reporting Items for Systematic Reviews and Meta-Analyses (PRISMA) 2020 recommendations [32]. A predefined review protocol was registered in the International Prospective Register of Systematic Reviews (PROSPERO) (CRD 395676). The aim was to identify and synthesize systematically all studies reporting original patient data on the association between lithium treatment, disturbances in calcium regulation, and hyperparathyroidism in adult patients, including studies describing surgical management and outcomes of LHPT.

### Eligibility criteria

Studies were eligible for inclusion if they: (1) included adult patients (≥ 18 years) with concurrent lithium treatment duration of at least six months; (2) reported disturbances in calcium homeostasis related to lithium therapy, defined primarily as hypercalcemia and/or hyperparathyroidism (hypocalcemia was retained at the search stage to capture all lithium-associated calcium abnormalities particularly in the postoperative setting, but is reported descriptively as a secondary finding); and (3) presented original human data, including case reports, case series, cohort studies, or clinical trials. No minimum sample size requirement was applied. Studies reporting parathyroid surgery in patients with suspected or confirmed LHPT were identified for additional surgical data extraction. Studies were excluded if they were review articles, narrative overviews, editorials, or commentaries without original data, conference abstracts lacking sufficient extractable patient information, animal or in vitro studies, studies not reporting calcium, parathyroid hormone (PTH), or parathyroid-related outcomes in relation to lithium therapy and studies involving pediatric populations or studies with lithium treatment durations less than six months.

### Information sources and search strategy

A comprehensive literature search strategy was developed. The following databases were searched: Ovid MEDLINE ALL, Embase, Cochrane Library, Web of Science Core Collection, and PsycInfo. The search strategy combined controlled vocabulary terms (MeSH and Emtree) with free-text terms related to lithium therapy and disturbances in calcium metabolism. The search was restricted to publications from 1973 to 2025 and to articles written in English, Danish, Norwegian, or Swedish. The complete database-specific search strategies are provided in the Supplementary Material.

### Outcomes

The primary outcome was the description of calcium regulation over time in lithium-treated adults, specifically plasma or serum calcium concentrations at baseline and at the last available follow-up after initiation of lithium therapy. Secondary outcomes included plasma or serum PTH concentrations at baseline and follow-up; prevalence or incidence of hypercalcemia in lithium-treated patients according to each study’s definitions; prevalence or incidence of LHPT; vitamin-D concentrations, where reported. For the surgical LHPT subset, additional outcomes included indications for surgery, preoperative biochemical profile and imaging findings, type and extent of parathyroidectomy, histological diagnosis, early postoperative biochemical status, and long-term outcomes including persistence, recurrence, reoperation and surgical complications. Rates of postoperative hypocalcemia were not consistently reported across studies and therefore could not be extracted systematically.

### Study selection and data extraction

Two reviewers (ADM and RAS) independently screened titles and abstracts after duplicate removal. Full texts of potentially eligible articles were independently assessed. Disagreements were resolved through discussion and consensus. Data extraction was performed in duplicate. Extracted variables included study characteristics, patient demographics, lithium exposure duration, biochemical parameters, and reported prevalence of hypercalcemia and LHPT. For surgical studies, additional variables included type of exploration, number of glands removed, histological diagnosis, and postoperative outcomes. Single-gland disease (SGD) was defined when authors reported a single adenoma or single pathological gland. Multiglandular disease (MGD) was defined when multiple enlarged glands were described, more than one gland was removed, or when hyperplasia was reported. Biochemical cure was assumed when authors described patients as normocalciemic or cured at follow-up.

### Data synthesis

Due to substantial heterogeneity in study design, patient populations, biochemical definitions, and duration of follow-up, data were synthesized using a descriptive approach. Formal meta-analysis was not feasible. Aggregated proportions should be interpreted as descriptive summaries of the published literature rather than precise effect estimates.

## Results

### Study selection

**Fig. 1:**
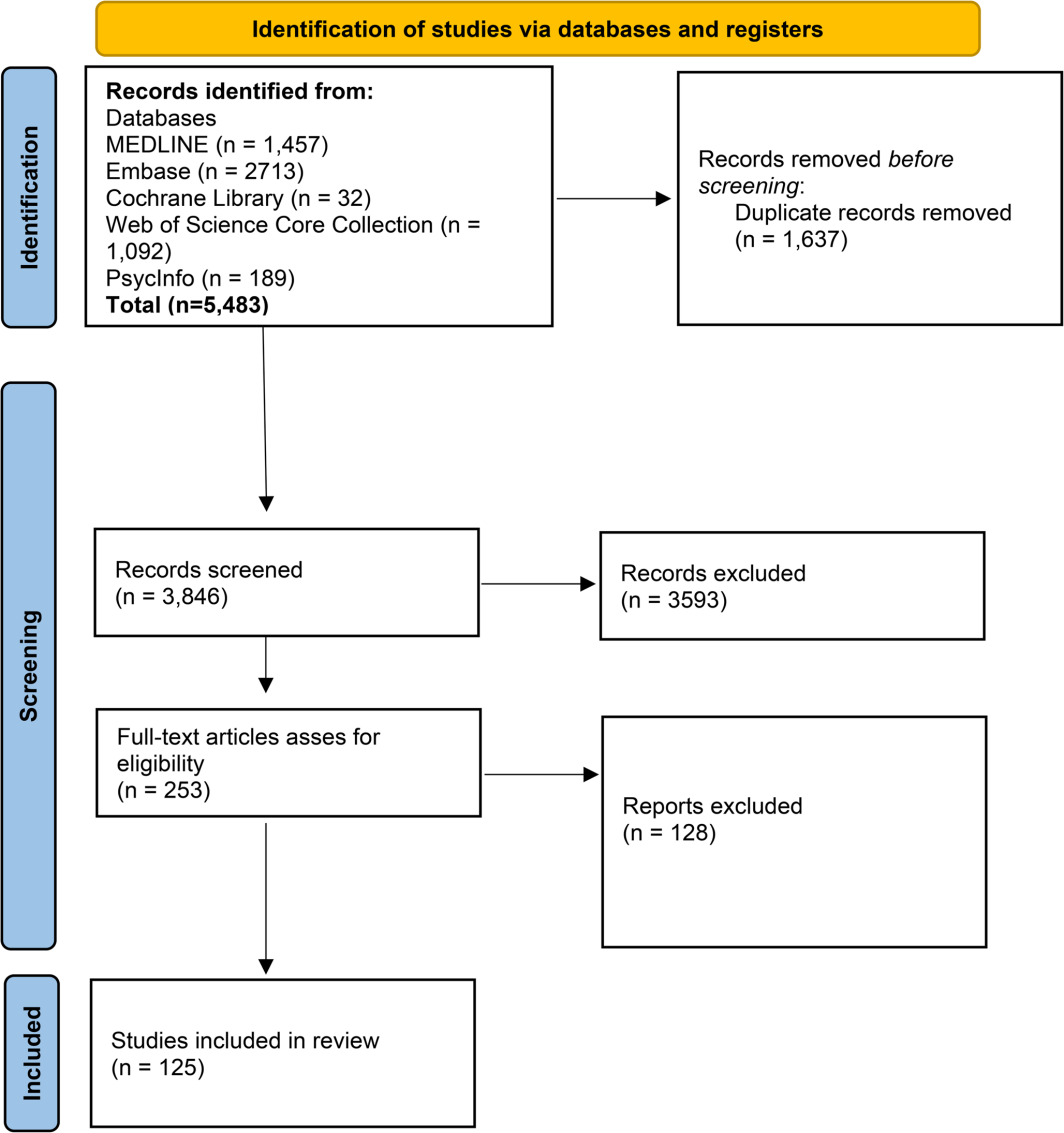
Systematic literature search (PRISMA flow chart)

The systematic search initially identified 5,483 records. After removal of duplicates, 3,846 unique records remained for title and abstract screening. Following this initial step, 253 articles were selected for full-text review. After application of the predefined eligibility criteria, 125 studies were included in the qualitative synthesis. The included publications consisted of observational cohort studies, cross-sectional studies, surgical case series, and individual case reports [11, 15–31, 33–104, 125–136]. The study selection process is summarized in Fig. 1.

### Study characteristics

The included studies were published between 1973 and 2025 and originated primarily from Europe, North America, and Australia. Study populations varied with respect to psychiatric diagnoses, duration of lithium exposure, and biochemical monitoring strategies. Duration of lithium therapy was reported inconsistently but frequently exceeded 10 years in cohorts evaluating LHPT.

### Prevalence of hypercalcemia and hyperparathyroidism

Studies reporting the prevalence of hypercalcemia and LHPT among lithium-treated patients are summarized in Table 1. Hypercalcemia was reported in 423 of 5,853 lithium-treated patients (7.2%) [15, 16, 33–39, 41–58]. Hyperparathyroidism was reported in 156 of 3,239 patients in whom parathyroid hormone (PTH) levels were measured (4.8%). Several studies reported cases of normocalcemic LHPT, characterized by elevated PTH levels despite normal serum calcium concentrations.

**Table 1:** Studies reporting levels of prevalence of hypercalcemia and hyperparathyroidism, respectively, based on study populations of lithium-treated patients. In addition, crude pooled prevalence is presented below

Study	No. Patients with Hypercalcaemia	Prevalence hypercalcaemia, %	No. Patients with HPT	Prevalence HPT, %
Albert ‘13	14/58	24.1	5/58	8.6
Albert ‘15			4/31	12.9
Awad ‘03			15/348	4.3
Bassilios ‘08	15/212	7		
Bendz ‘96			8/142	5.6
Boivin ‘23	30/70	17.6		
Carchman ‘08			16/1207	1.3
Christiansen ‘76	12/96	12.5		
Dillenberger ‘20	5/557*	0.9		
Dineen ‘17	37/503	6.4	4/503**	0.8
Hayes ‘16	10/2148	0.47		
Holroyd ‘94	1/114	0.88		
Jones ‘09	5/43	11.6		
Kallner ‘94	3/207	6		
Kuman ‘19			5/87	5.7
Lally ‘13	15/333	5.3	3/333	1
Linder ‘93	19/110	20.9		
Lozano ‘16			3/32	9
Luby ‘03			3/14	21
McIntosh ‘87	2/61	3.3		
Meehan ‘15			77/423	18
Meehan ‘18	87/563	26		
Meehan ‘20	102/297	34		
Nordenström ‘94	14/26	54	5/26	19
Oliveira ‘14			8/35	23
Twigt ‘13	49/314	15.6		
van Melick ‘13	3/111	2.7		
TOTAL	423/5853	7.2	156/3239	4.8

*5/557 were operated for HPT

**16/503 (2.8%) had PTH measurements

### Surgical findings

Operative findings in surgical studies reporting ≥ 10 lithium-treated patients having undergone parathyroidectomy are summarized in Table 2 [16, 20–22, 28, 35, 39, 59, 73]. Among these, the largest surgical series included 71 patients undergoing 78 operations [21]. The median duration of lithium therapy before surgery ranged from approximately 9.8 to 21 years. Both single-gland disease and multiglandular disease were reported. In the largest surgical series, 32 patients had single-gland adenomas, 2 patients had double adenomas, and 37 patients had multiglandular hyperplasia [21]. Across the surgical cohorts, the reported proportion of multiglandular disease ranged from approximately 25% to more than 83% of operated patients [15–22, 24–31].

**Table 2:** Study characteristics of surgical studies reporting ≥ 10 lithium-treated patients having undergone parathyroidectomy

Study (Year)	*N*	No of operations	No of Glands identified	No of Glands removed	Weight of resected glands (g)	Morphology SGD/MGD	Cure at initial operation %	Follow-up years	Recurrence no of pts
Abdullah (1999)	11	12	42	18.5	0.74	Adenoma & hyperplasia; SGD*	100%	2–30	2
Awad (2003)	15	15	60	19.5	-	14 had SGD, 1 had MGD	100%	-	1
Carchman (2008)	16	16	33	20	Range 0.036–4.283	12 had SGD; Adenoma. 2 had MGD; Adenoma, 2 nodular hyperplasia	100%	4.1	-
Hundley (2005)	12	12	44	20.25	Range 0.06–2.1	6 had MGD; others not stated	25%	Follow up was done, but no timeframe	-
Järhult (2010)	71	78	134	134	Range 0.354–1.535	32 had SGD; Adenoma. 2 had MGD; Adenoma, 37 had Hyperplasia	58%	0.5–6.3	6
Lally (2013)	15	1	-	-	-	1 had MGD; pathology not stated	-	-	-
Marti (2012)	27	16	64	64	-	10 had SGD (Adenoma). 15 had MGD (Adenoma and Hyperplasia)	“Some yes, some no”.	0.5	-
McIntosh (1987)	61	-	1	-	-	1 had SGD; Adenoma	-	-	-
Meehan (2020)	29	33	Group A; Median 3 (1–3) Group B; Median 3 (2–4)	70	-	Group A; 5 normal, 15 hyperplasia, 1 nodular hyperplasia, 4 adenoma Group B; 3 normal, 32 nodular hyperplasia, 4 adnoma	34%	Group A; median mths 34 (24–308) Group B; median mths	9
Presne (2003)	74	3	-	3	-	2 had SGD; Adenoma. 1 SGD; Adenoma superimposed on hyperplasia.	-	-	-
Skandarajah (2011)	15	15	-	33	Range 0.67675 (0.050-8.0)	4 had SGD; Adenoma. 11 had MGD; Hyperplasia.	73%	2.33 (1.16–6.16)	-
Stancer (1989)	19	3	12	6	-	SGD; Adenoma Hyperplasia	100%	-	-
Twight (2013)	49	2	-	-	-	-	-	-	-
Wade (2013)	19	19	Not stated exactly how many they identified (10 underwent bilat. expl. 3 unilat. 6 minimal. invasive)	-	SGD; Median 0.590 (0.134–6.750) MGD; 0.296 (0.145–2.170)	13 had SGD; Adenoma. 6 had MGD.	100%	1.58 (0.16–9.83)	-

### Extent of surgical exploration and number of glands resected

Bilateral neck exploration was performed in the majority of patients in surgical series. Awad et al. reported bilateral exploration in all 15 patients [35]; Järhult et al. bilateral exploration in 69 of 71 patients [21]; and Hundley et al. bilateral exploration in 10 of 12 patients [20]. Other studies reported a combination of bilateral exploration, unilateral exploration, or minimally invasive parathyroidectomy depending on preoperative localization findings. When multiglandular disease was encountered, removal of multiple glands was common. In the cohort of Järhult et al., 12 patients had three glands removed and 8 patients had three and a half glands removed [21]. In the study by Skandarajah et al., four patients underwent removal of three glands and two patients underwent removal of four glands [28].

### Postoperative outcomes

Postoperative biochemical outcomes were reported in most surgical studies. In the largest cohort reported by Järhult et al., long-term biochemical cure was achieved in a substantial proportion of patients following parathyroidectomy, with outcomes appearing similar among patients with adenomatous and hyperplastic disease during follow-up ranging from 6 months to 6.3 years [21]. In the cohort of Carchman et al., all 16 patients were reported to be biochemically cured, with a follow-up range of 5 to 50 months [39]. Meehan et al. report a series of 29 patients, 10 patients were cured, 5 had persistent disease, 5 had normocalcaemic HPT, and 9 developed recurrent hyperparathyroidism, with a median follow-up of 34 months [16]. Across surgical cohorts, recurrence of hyperparathyroidism was reported in several studies [16, 20–22, 28, 35, 59]. Postoperative hypocalcemia was not reported consistently enough across included surgical studies to permit pooled or systematic extraction.

## Discussion

This systematic review provides a comprehensive synthesis of lithium-associated hyperparathyroidism (LHPT), demonstrating that hypercalcemia occurs in 7.2% and LHPT in 4.8% of lithium-treated patients, representing an 8–10 fold increased risk compared to the general population prevalence of 0.5% [8, 14, 15]. A critical finding is the substantially higher prevalence of multiglandular disease (25–83%, estimate 51%) in LHPT compared to sporadic PHPT (15–20%), with important implications for surgical planning [20, 21, 28, 105].

**Fig. 2:**
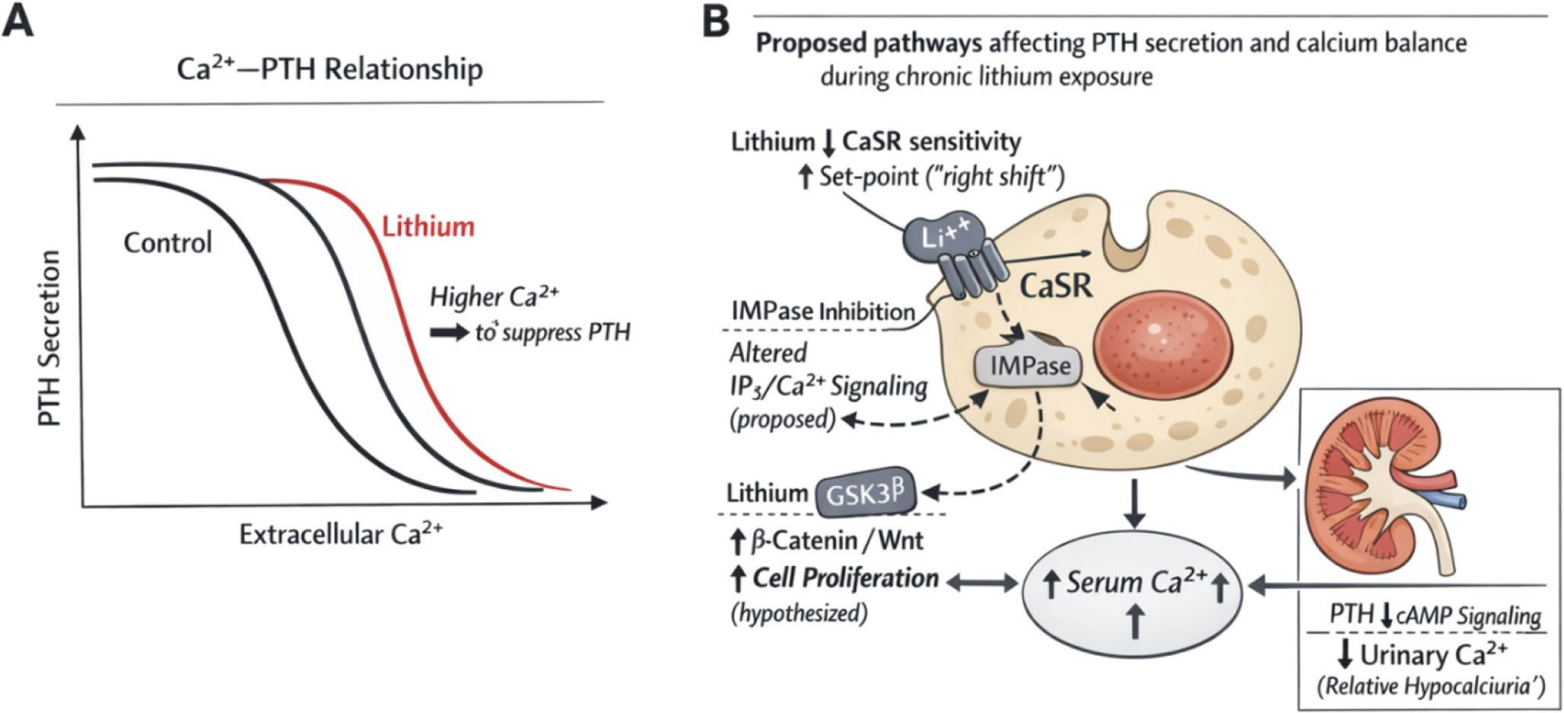
Lithium-associated disruption of calcium homeostasis: set-point shift and proposed intracellular mechanisms. **A** Conceptual Ca²⁺–PTH response curve illustrating a rightward shift of the calcium set-point during lithium exposure, such that higher extracellular calcium is required to suppress PTH secretion. **B** Schematic overview of pathways by which chronic lithium may increase PTH secretion and contribute to hypercalcemia. Lithium is associated with reduced functional sensitivity of the calcium-sensing receptor (CaSR) (“right shift”). Additional intracellular mechanisms have been proposed, including inhibition of inositol monophosphatase (IMPase) with downstream modulation of phosphoinositide/IP₃ signaling and intracellular Ca²⁺ concentration, and inhibition of GSK3β with activation of β-catenin/Wnt pathways that may contribute to parathyroid growth. Renal effects (including altered cAMP signaling) may contribute to relative hypocalciuria. Solid arrows indicate established physiological relationships; dashed arrows indicate proposed mechanisms

Lithium-associated disturbances in calcium metabolism arise primarily from alterations in parathyroid gland physiology. The calcium-sensing receptor (CaSR) on parathyroid chief cells plays a central role in calcium homeostasis by detecting extracellular calcium concentrations and modulating parathyroid hormone (PTH) secretion (Fig. 2A). Lithium interacts with the CaSR, producing a rightward shift in the calcium–PTH set-point, thereby requiring higher serum calcium concentrations to suppress PTH secretion [9, 15, 106]. This altered set-point results in relative PTH hypersecretion at any given calcium level, leading to the characteristic biochemical profile of hypercalcemia with inappropriately normal or elevated PTH concentrations. Beyond receptor-mediated effects, lithium inhibits inositol monophosphatase and glycogen synthase kinase-3β (GSK-3β), a key regulator of the Wnt/β-catenin signaling pathway [107–109]. Inhibition of GSK-3β leads to β-catenin accumulation and activation of genes implicated in cellular proliferation and reduced apoptosis, potentially promoting parathyroid gland growth (Fig. 2B).

The chronic nature of lithium exposure appears to be an important determinant of parathyroid pathology. Prolonged stimulation through the altered calcium set-point, combined with the proliferative effects of GSK-3β inhibition, may promote adenoma formation in susceptible individuals while diffuse hyperplasia develops in others. This dual pathogenic mechanism likely accounts for the heterogeneous pathological patterns reported in lithium-associated hyperparathyroidism, encompassing both solitary adenomas and multiglandular hyperplasia [20–22, 25, 26, 28, 30, 59, 110]. The observation that multiglandular disease is substantially more common in LHPT than in sporadic primary PHPT supports the hypothesis that lithium exerts a diffuse, systemic effect on all parathyroid glands rather than promoting focal neoplastic transformation in a single gland. The variable latency between initiation of lithium therapy and development of clinically apparent LHPT—ranging from months to decades—suggests that individual susceptibility factors may modulate the risk and timing of parathyroid dysfunction. Recent data suggest that serum lithium concentrations above 0.52 mEq/L are associated with increased risk of LHPT, indicating a potential dose-response relationship [20, 21, 25, 30, 59, 111, 112] .

### Prevalence and clinical implications

In the present systematic review, hypercalcemia was reported in 7.2% of lithium-treated patients, while LHPT was confirmed in 4.8% (Table 1) [15, 16, 33–39, 41–58]. These estimates with an approximately 8–10 fold increased HPT risk compared to the general population, suggest that lithium is an important risk factor for HPT. Several studies reported mild biochemical abnormalities or isolated elevations in parathyroid hormone levels without concomitant hypercalcemia, suggesting that disturbances in calcium regulation may develop gradually during prolonged lithium exposure [11, 21, 54, 113], while others could not confirm this association [33, 35, 59].

Recent literature has emphasized inadequate screening practices in lithium-treated patients. In one large cohort, 25 of lithium-treated patients received no further evaluation despite documented hypercalcemia [15]. Current recommendations support baseline calcium measurement before initiating lithium therapy, followed by annual monitoring, and addition, if calcemic dysfunction is detected, careful monitoring should be implemented. Takano et al. identified that serum lithium concentrations above 0.52 mEq/L are associated with increased risk of LHPT, suggesting that patients with higher lithium levels may warrant closer monitoring [114].

Vandermeulen et al. recently published a systematic review and meta-analysis on lithium-associated hypercalcemia and hyperparathyroidism [112]. Their analysis reported a pooled prevalence of 51% for multiglandular disease in LHPT, consistent with the range of 25–83% observed in our surgical series. Our review complements this meta-analysis by providing a comprehensive narrative synthesis of the surgical literature, including detailed analysis of operative strategies, extent of resection, and long-term outcomes. Importantly, several important patterns emerge from the surgical literature. Most surgical series are from the 2000s onward, indicating relatively modern surgical care [15, 16, 19–22, 24, 26–30, 39, 62, 65–67, 69, 71–73, 114] with Järhult et al. study being the largest and most comprehensive one [21]. Approximately two-thirds of surgical patients are women, similar to sporadic PHPT. Age appears to influence outcomes, as single-gland disease was more common in younger patients, though this observation requires further validation [39, 73]. The paucity of long-term follow-up data across studies limits definitive conclusions with regards to recurrence rates. In addition, LHPT develops progressively with increasing duration of lithium treatment [15, 16, 21, 54, 115]. However, the majority (> 60%) of patients treated with lithium for several decades do not develop LHPT, suggesting individual susceptibility factors that modulate LHPT risk. Among those who underwent surgery, lithium duration appears to be an important factor, with longer exposure associated with higher risk of persistent and recurrent disease.

A key finding of this review is the marked variability in reported prevalence LHPT, which is likely driven by methodological rather than biological differences. Study populations differ substantially, ranging from unselected lithium-treated cohorts to highly selected endocrine or surgical referral populations, limiting direct comparison of prevalence estimates [21, 33, 48, 54, 56, 59]. Nevertheless, definitions of LHPT are inconsistent, with some studies requiring both hypercalcemia and elevated PTH, while others include isolated biochemical abnormalities or single measurements [33, 51, 54]. Currently, there is no consensus definition of LHPT beyond that proposed by our group in 2015 [54], though expert opinion has formulated management guidelines [12, 116].

In addition, biochemical thresholds varied between studies. Some used albumin-corrected calcium, others total calcium only, whereas reference ranges for calcium and PTH differed between laboratories and over time, particularly in older studies. Since many patients cluster near the upper reference limit, small differences in assay characteristics can shift classification from normal to abnormal. These differences in biochemical thresholds, laboratory methods, apart from differences in patient characteristics, such as age and lithium exposure further influence the reported prevalence estimates [8, 30, 96, 108, 111, 117–121]. Overall, when restricted to larger and more comparable cohorts, prevalence estimates appear to converge, suggesting that LHPT affects a minority but clinically relevant proportion of lithium-treated patients [15, 16, 33, 36, 38, 41, 43, 49, 50, 54, 56, 57] (Table 1). Future studies should adopt standardized diagnostic criteria for LHPT and harmonized biochemical thresholds for calcium and PTH, ideally using consistent assay methods and reference ranges. Such standardization would reduce methodological variability in reported prevalence estimates and facilitate more meaningful comparison of outcomes across different populations and study designs.

With respect to lithium exposure, studies included patients with variable durations of lithium treatment. Longitudinal studies demonstrate progressive increases in calcium and PTH with prolonged lithium exposure. In addition, older patients with long-term use showed higher proportions of LPTH. Importantly, the background epidemiological risk of sporadic PHPT in lithium treated patients and secondary causes of PTH elevation, including renal impairment and vitamin D deficiency, also differ markedly between younger psychiatric populations and older multimorbid ones.

### Multiglandular disease and surgical planning

A key finding of this review is the substantially higher prevalence of multiglandular disease in LHPT compared to sporadic PHPT. When interpreting the reported rates of persistent and recurrent hyperparathyroidism, it is important to recognize that many historical series included patients treated with focused or unilateral exploration despite a relatively high underlying prevalence of multiglandular disease in LHPT. In such settings, limited exploration may leave occult multiglandular disease untreated and thereby increase the apparent recurrence and persistence rates, independently of any intrinsically more aggressive disease biology.

While sporadic PHPT demonstrates multiglandular involvement in approximately 15–20% of cases, our surgical series showed rates ranging from 25% to over 83% [15–22, 24, 26–31]. This represents an approximately 3-fold increased risk of multiglandular disease and has critical implications for surgical planning. The 2016 American Association of Endocrine Surgeons (AAES) guidelines state that in LHPT, the surgical approach may be bilateral exploration or minimally invasive parathyroidectomy guided by imaging and intraoperative PTH monitoring [122]. However, this recommendation is weak based on low-quality evidence, and therefore bilateral exploration remains the preferred strategy especially when preoperative imaging is discordant or non-localizing, when there is high suspicion of multiglandular disease, or when intraoperative PTH monitoring is unavailable. Indeed, the higher prevalence of multiglandular disease reported in several surgical series, particularly those of Järhult et al. and Meehan et al., has led many surgeons to advocate a lower threshold for bilateral neck exploration than would typically be adopted in sporadic PHPT [15, 21]. However, several studies demonstrated favorable outcomes with focused parathyroidectomy when preoperative localization studies clearly identify a single pathological gland. Wade et al. demonstrated that of 19 patients with lithium exposure, 13 (68%) had single-gland disease, and minimally invasive parathyroidectomy with intraoperative PTH monitoring was successfully performed [73]. Norlén et al. reported favorable long-term outcomes using a predominantly focused surgical strategy guided by preoperative localization and intraoperative parathyroid hormone monitoring, with a cumulative recurrence rate of 16% after 10 years and durable biochemical cure in the majority of patients [26]. These findings suggest that a substantial subset of patients with LHPT harbor clinically relevant single-gland disease and can be treated successfully with a targeted approach. Taken together, the available evidence supports an individualized operative strategy in which focused parathyroidectomy may be appropriate in patients with concordant localization studies and satisfactory intraoperative PTH decline, while surgeons should maintain a lower threshold for bilateral neck exploration in patients with discordant imaging, prolonged lithium exposure, or intraoperative findings suggestive of multiglandular disease.

A major limitation of the current literature is the lack of detailed reporting on preoperative localization studies. Most surgical series provide limited information regarding imaging modality, localization accuracy, concordance between ultrasound and sestamibi scintigraphy, use of advanced imaging techniques such as 4D-CT, or the relationship between imaging findings and final pathology. Consequently, it remains unclear whether the increased prevalence of multiglandular disease in LHPT significantly reduces the accuracy of conventional localization studies compared with sporadic PHPT, as detailed imaging and localization outcomes are inconsistently reported across surgical series [16, 22, 26, 39, 73]. This knowledge gap has important implications for surgical planning because reliable localization is fundamental to the success of focused parathyroidectomy. Furthermore, few studies reported intraoperative PTH responses in sufficient detail to determine whether standard intraoperative PTH criteria perform equally well in patients with lithium-associated disease. Given the frequent coexistence of lithium-related renal dysfunction and altered calcium–PTH homeostasis, the interpretation of intraoperative PTH kinetics may be more complex than in sporadic PHPT [8, 9]. Future studies should systematically report preoperative imaging findings, intraoperative PTH responses, operative strategy, and long-term outcomes to better identify which patients can safely undergo focused surgery and which are more likely to benefit from bilateral neck exploration.

### Long-term outcomes and recurrence

Long-term surgical outcomes for LHPT are characterized by higher rates of persistent and recurrent disease compared to sporadic PHPT; however, these rates must be interpreted in the context of variable operative strategies, as limited or selective exploration in patients with multiglandular disease can contribute substantially to persistence and recurrence. In the largest cohort reported by Järhult et al., the rate of persistent and recurrent hyperparathyroidism was 42% regardless of histopathological diagnosis [21]. In contrast, Norlén et al. reported a 10-year cumulative recurrence rate of 16% (95% CI 2–29%), with a long-term cure rate exceeding 80% [26]. The risk of recurrence is particularly elevated in patients with longer duration of lithium exposure and older age at surgery. Permanent hypoparathyroidism occurs in up to 13% of cases when multiple glands are removed. The literature emphasizes the need to balance the risk of recurrence against the risk of permanent hypoparathyroidism.

### Limitations

Several limitations should be acknowledged. The majority of included studies were retrospective observational studies or small case series, limiting the strength of the available evidence. There was substantial heterogeneity across studies with respect to patient populations, biochemical definitions, and duration of lithium exposure. Many studies did not report complete biochemical data or detailed information regarding lithium exposure. Despite the large number of included studies, substantial heterogeneity in study design, patient selection, biochemical definitions, and outcome reporting limited meaningful quantitative pooling. We therefore performed a descriptive synthesis aimed at identifying consistent patterns across the available literature rather than deriving potentially misleading summary estimates. The surgical literature consisted primarily of single-institution case series, which may introduce referral bias, as patients undergoing surgery likely represent a selected subgroup with more severe biochemical abnormalities. Long-term follow-up data were inconsistently reported, making it difficult to determine the true rate of recurrent or persistent disease following surgery. In addition, the lack of consistent reporting of postoperative hypocalcemia limits assessment of this complication and prevents meaningful comparison across surgical series. Overall, formal meta-analysis was not performed due to heterogeneity. Publication bias is likely, as surgical series may overrepresent complex cases. Finally, limited data exist on non-surgical management options such as cinacalcet. Moreover, the surgical literature in this field consists largely of single-institution case series with heterogeneous reporting of preoperative imaging, intraoperative PTH monitoring, and long-term follow-up, which limits the ability to draw firm conclusions regarding the optimal operative strategy and the true rates of persistent and recurrent disease in LHPT. Taken together, these factors mean that the strength of evidence supporting specific management strategies is modest, and our clinical recommendations should be interpreted accordingly.

### Clinical significance and future directions

The clinical significance of LHPT for individual patients remains incompletely characterized and warrants further investigation. While the biochemical abnormalities are well-documented, the impact on bone health, renal function, cardiovascular outcomes, and quality of life in lithium-treated patients with LHPT compared to those without calcium dysregulation is poorly understood [15, 16].

Critically, the clinical significance of mild hypercalcemia in lithium-treated patients remains unknown. Many patients demonstrate calcium levels in the range of 2.60–2.75 mmol/L (10.4–11.0 mg/dL) with mildly elevated or inappropriately normal PTH, yet the long-term consequences of this chronic mild hypercalcemia have not been systematically studied. It is unclear whether such patients experience accelerated bone loss, increased cardiovascular risk, progressive renal impairment, or cognitive decline compared to normocalcemic lithium-treated controls. The threshold at which intervention becomes beneficial—whether through surgery, medical management, or lithium discontinuation—remains undefined for patients with mild biochemical abnormalities. The association between hypercalcemia and mood symptoms is well-documented, raising the possibility that correction of calcium abnormalities might improve psychiatric outcomes, though this hypothesis requires prospective evaluation. Conversely, the psychiatric risks of parathyroidectomy in this vulnerable population, including potential mood destabilization during the perioperative period, have not been adequately characterized.

A randomized controlled trial comparing surgical versus medical management (including cinacalcet) versus observation in LHPT could provide valuable evidence regarding optimal treatment strategies and patient-centered outcomes. Such a trial would need to address not only biochemical endpoints but also skeletal health, renal function, psychiatric stability, cognitive function, and quality of life measures across the spectrum of disease severity, particularly in patients with mild hypercalcemia.

### Clinical implications

Based on the currently available evidence, the following management considerations may be useful in clinical practice. 

*Monitoring*: Annual serum calcium measurement may be considered in all lithium-treated patients, with PTH assessment if hypercalcemia develops. Patients with serum lithium concentrations above 0.52 mEq/L may warrant more frequent monitoring, although this recommendation is based on retrospective data and should be interpreted cautiously [114, 123, 124]. 

Surgical planning: Given the prevalence of approximately 51% of multiglandular disease, preoperative imaging should be interpreted cautiously. When imaging suggests single-gland disease and intraoperative PTH monitoring is available, focused parathyroidectomy can be considered in carefully selected patients. However, surgeons may reasonably maintain a low threshold for bilateral exploration, particularly in patients with non-localizing imaging, prolonged lithium exposure exceeding 10 years, or intraoperative findings suggestive of multiglandular involvement, acknowledging that these suggestions are based on retrospective series and expert opinion rather than prospective trials. 

Postoperative management: Long-term biochemical follow-up is advisable given recurrence rates of approximately 16% at 10 years in published series. Continued lithium therapy appears to be feasible after successful parathyroidectomy in many patients, but the decision to continue or discontinue lithium should be individualized and made in collaboration with the treating psychiatrist, recognizing the limited evidence base.

Overall, these clinical recommendations should be regarded as suggested management considerations derived from retrospective observational data and expert opinion, and they require prospective validation and formal consensus development.

## Conclusion

Lithium-associated hyperparathyroidism is a distinct form of primary hyperparathyroidism characterized by heterogeneous gland pathology and a greater propensity for multiglandular involvement than sporadic disease. Although focused parathyroidectomy can achieve durable biochemical cure in carefully selected patients with concordant localization studies and appropriate intraoperative adjuncts, the available evidence supports a lower threshold for bilateral neck exploration because of the increased prevalence of multiglandular disease and the risk of persistent or recurrent hyperparathyroidism. Current evidence is limited by the retrospective nature of most studies and the paucity of standardized imaging and intraoperative PTH data. Prospective multicenter studies are needed to refine patient selection, optimize operative strategy, and define the long-term outcomes of surgical and medical treatment in this increasingly recognized endocrine disorder, thereby providing a stronger evidence base for future practice recommendations.

## Supplementary Information

Supplementary Material 1.

## Data Availability

All data analyzed in this systematic review were obtained from previously published studies cited in the manuscript. No new datasets were generated. The extracted data supporting the findings of this review are available from the corresponding author upon reasonable request.
