# Supplementary material for: Lithium-associated hyperparathyroidism: a systematic review of prevalence and surgical outcomes
Source: Langenbecks Arch Surg. 2026 Aug 17;411(1):216. doi: 10.1007/s00423-026-04200-5 (PMC13481389; doi:10.1007/s00423-026-04200-5)
Supplement: Supplementary file 1 [file 423_2026_4200_MOESM1_ESM.docx]

The total number of references was 5,483 before deduplication and 3,846 after deduplication.

Database(s): Ovid MEDLINE(R)

| Söktermer | | | Antal träffar |
| --- | --- | --- | --- |
| Lithium | | | |
|  | 1. | Lithium/ or Lithium Chloride/ or Lithium Compounds/ or Lithium Carbonate/ or Bromides/ | 36,674 |
|  | 2. | (lithium* or lihionit or eskalith or lithotabs or lithane or dilithium or lithobid or lithonate or "CP-15,467-61" or "CP15,46761" or micalith or "NSC-16895" or NSC16895 or priadel or "Quilinorm-retard" or Quilinormretard or camcolit or candamide or carbolit or carbolith or carbolitium or ceglution or contemnol or duralith or hypnorex or lentolith or licab or licarb or licarbium or lidin or limas or linthane or liskonium or liskonum or litheum or lithicarb or lithiumcarbonate or lithmax or lithobid or lithocap or lithocarb or lithionate or litilent or litocarb or maniprex or neurolepsin or phanate or plenur or quilonorm or teralithe or teralite or "li-liquid" or cibalith or litarex or mesin or lithium sulphate or Lithiophor or Lithiofor).mp. [mp=title, book title, abstract, original title, name of substance word, subject heading word, floating sub-heading word, keyword heading word, organism supplementary concept word, protocol supplementary concept word, rare disease supplementary concept word, unique identifier, synonyms, population supplementary concept word, anatomy supplementary concept word] | 70,643 |
|  | 3. | 1 or 2 | 76,597 |
| Hyper- or hypoparathyroidism, hyper- or hypocalcemia | | | |
|  | 4. | exp Hyperparathyroidism/ or exp Hypoparathyroidism/ or exp Parathyroid Hormone/ | 55,615 |
|  | 5. | (Hyperparathyroid* or "hyper parathyroid*" or "hyperparathyroid crisis" or hyperparathyroidea or hyperparathyroidosis or "hypertrophic parathyroid" or "parathyroid gland hypertrophy" or "parathyroid hyperfunction" or "parathyroid hypertrophy" or hypoparathyroid* or "Hypo parathyroid*" or hypoparathyreosis or hypoparathyroidy or parathyroid hypofunction or parathyroid insufficiency or Parathyroid or Parathormone or Parathyrin or Natpara or PTH or parathorm or parathormon or "parathyroid hormone*").ti,ab,kf. | 74,922 |
|  | 6. | Hypercalcemia/ or exp Hypocalcemia/ or Hypercalciuria/ | 23,680 |
|  | 7. | (hypercalcemi* or "milk-alkali syndrome" or hypercalcaemi* or hypercalciema or Hypocalcemi* or hypocalcaemi* or hypocalcinemia or hypocalciuria or hypercalciuria).ti,ab,kf. | 35,761 |
|  | 8. | exp Calcium/ | 282,083 |
|  | 9. | ((calcium or "Ca2+") adj7 (increas* or decreas* or high* or low* or level* or drop* or down or regulat* or dysregulat* or abnormal*)).ab,kf,ti. | 179,326 |
|  | 10. | (increas* or decreas* or high* or low* or level* or drop* or down or regulat* or dysregulat* or abnormal*).ab,kf,ti. | 17,040,511 |
|  | 11. | 8 and 10 | 192,381 |
|  | 12. | 4 or 5 or 6 or 7 or 9 or 11 | 362,224 |
| Combined sets | | | |
|  | 13. | 3 and 12 | 1,630 |
| Limits | | | |
|  | 14. | limit 13 to yr="1973 -Current" | 1,568 |
|  | 15. | limit 14 to (danish or english or norwegian or swedish) | 1,457 |

Cochrane 2023-03-09

| Söktermer | | | Antal träffar |
| --- | --- | --- | --- |
| Lithium | | | |
|  | #1 | MeSH descriptor: [Lithium] this term only | 716 |
|  | #2 | MeSH descriptor: [Lithium Chloride] this term only | 28 |
|  | #3 | MeSH descriptor: [Lithium Compounds] this term only | 185 |
|  | #4 | MeSH descriptor: [Lithium Carbonate] this term only | 462 |
|  | #5 | ((lithium* or lihionit or eskalith or lithotabs or lithane or dilithium or lithobid or lithonate or "CP-15,467-61" or "CP15,46761" or micalith or "NSC-16895" or NSC16895 or priadel or "Quilinorm-retard" or Quilinormretard or camcolit or candamide or carbolit or carbolith or carbolitium or ceglution or contemnol or duralith or hypnorex or lentolith or licab or licarb or licarbium or lidin or limas or linthane or liskonium or liskonum or litheum or lithicarb or lithiumcarbonate or lithmax or lithobid or lithocap or lithocarb or lithionate or litilent or litocarb or maniprex or neurolepsin or phanate or plenur or quilonorm or teralithe or teralite or "li-liquid" or cibalith or litarex or mesin or lithium sulphate or Lithiophor or Lithiofor)):ti,ab,kw | 3,345 |
|  | #6 | #1 or #2 or #3 or #4 or #5 | 3,345 |
| Hyper- or hypoparathyroidism, hyper- or hypocalcemia | | | |
|  | #7 | MeSH descriptor: [Hyperparathyroidism] explode all trees | 754 |
|  | #8 | MeSH descriptor: [Hypoparathyroidism] explode all trees | 130 |
|  | #9 | MeSH descriptor: [Parathyroid Hormone] explode all trees | 1,829 |
|  | #10 | ((Hyperparathyroid* or "hyper parathyroid*" or "hyperparathyroid crisis" or hyperparathyroidea or hyperparathyroidosis or "hypertrophic parathyroid" or "parathyroid gland hypertrophy" or "parathyroid hyperfunction" or "parathyroid hypertrophy" or hypoparathyroid* or "hypo parathyroid*" or hypoparathyreosis or hypoparathyroidy or parathyroid hypofunction or parathyroid insufficiency or Parathyroid or Parathormone or Parathyrin or Natpara or PTH or parathorm or parathormon or parathormone or "parathyroid hormone*")):ti,ab,kw | 6,594 |
|  | #11 | MeSH descriptor: [Hypercalcemia] this term only | 246 |
|  | #12 | MeSH descriptor: [Hypocalcemia] explode all trees | 225 |
|  | #13 | MeSH descriptor: [Hypercalciuria] this term only | 48 |
|  | #14 | ((hypercalcemi* or "milk-alkali syndrome" or hypercalcaemi* or hypercalciema or Hypocalcemi* or hypocalcaemi* or hypocalcinemia or hypocalciuria or hypercalciuria)):ti,ab,kw | 2,964 |
|  | #15 | MeSH descriptor: [Calcium] explode all trees | 4,034 |
|  | #16 | (((calcium or "Ca2+") NEAR/7 (increas* or decreas* or high* or low* or level* or drop* or down or regulat* or dysregulat* or abnormal*))):ti,ab,kw | 8,038 |
|  | #17 | ((increas* or decreas* or high* or low* or level* or drop* or down or regulat* or dysregulat* or abnormal*)):ti,ab,kw | 1,176,061 |
|  | #18 | #15 and #17 | 3,489 |
|  | #19 | #7 or #8 or #9 or #10 or #11 or #12 or #13 or #14 or #16 or #18 | 15,255 |
| Combined sets | | | |
|  | #20 | #6 and #19 |  |
| Limits | | | |
|  | #21 | #6 and #19 with Cochrane Library publication date Between Jan 1973 and Mar 2023 | 32 |

Embase via embase.com 2023-03-09

| Söktermer | | | Antal träffar |
| --- | --- | --- | --- |
| Lithium | | | |
|  | 1. | 'lithium'/exp OR 'lithium citrate'/exp OR 'lithium carbonate'/exp OR 'lithium chloride'/exp OR 'lithium derivative'/exp OR 'bromide'/de | 91,910 |
|  | 2. | lithium*:ab,kw,ti,tn OR lihionit:ab,kw,ti,tn OR eskalith:ab,kw,ti,tn OR lithotabs:ab,kw,ti,tn OR lithane:ab,kw,ti,tn OR dilithium:ab,kw,ti,tn OR lithonate:ab,kw,ti,tn OR 'cp-15,467-61':ab,kw,ti,tn OR 'cp15,46761':ab,kw,ti,tn OR micalith:ab,kw,ti,tn OR 'nsc-16895':ab,kw,ti,tn OR nsc16895:ab,kw,ti,tn OR priadel:ab,kw,ti,tn OR 'quilinorm-retard':ab,kw,ti,tn OR quilinormretard:ab,kw,ti,tn OR camcolit:ab,kw,ti,tn OR candamide:ab,kw,ti,tn OR carbolit:ab,kw,ti,tn OR carbolith:ab,kw,ti,tn OR carbolitium:ab,kw,ti,tn OR ceglution:ab,kw,ti,tn OR contemnol:ab,kw,ti,tn OR duralith:ab,kw,ti,tn OR hypnorex:ab,kw,ti,tn OR lentolith:ab,kw,ti,tn OR licab:ab,kw,ti,tn OR licarb:ab,kw,ti,tn OR licarbium:ab,kw,ti,tn OR lidin:ab,kw,ti,tn OR limas:ab,kw,ti,tn OR linthane:ab,kw,ti,tn OR liskonium:ab,kw,ti,tn OR liskonum:ab,kw,ti,tn OR litheum:ab,kw,ti,tn OR lithicarb:ab,kw,ti,tn OR lithiumcarbonate:ab,kw,ti,tn OR lithmax:ab,kw,ti,tn OR lithobid:ab,kw,ti,tn OR lithocap:ab,kw,ti,tn OR lithocarb:ab,kw,ti,tn OR lithionate:ab,kw,ti,tn OR litilent:ab,kw,ti,tn OR litocarb:ab,kw,ti,tn OR maniprex:ab,kw,ti,tn OR neurolepsin:ab,kw,ti,tn OR phanate:ab,kw,ti,tn OR plenur:ab,kw,ti,tn OR quilonorm:ab,kw,ti,tn OR teralithe:ab,kw,ti,tn OR teralite:ab,kw,ti,tn OR 'li-liquid':ab,kw,ti,tn OR cibalith:ab,kw,ti,tn OR litarex:ab,kw,ti,tn OR mesin:ab,kw,ti,tn OR 'lithium sulphate':ab,kw,ti,tn OR lithiophor:ab,kw,ti,tn OR lithiofor:ab,kw,ti,tn | 65,764 |
|  | 3. | 1 OR 2 | 114,118 |
| Hyper- or hypoparathyroidism, hyper- or hypocalcemia | | | |
|  | 4. | 'hyperparathyroidism'/exp OR 'hypoparathyroidism'/exp OR 'parathyroid hormone'/exp | 101,414 |
|  | 5. | hyperparathyroid*:ab,kw,ti OR 'hyper parathyroid*':ab,kw,ti OR 'hyperparathyroid crisis':ab,kw,ti OR hyperparathyroidea:ab,kw,ti OR hyperparathyroidosis:ab,kw,ti OR 'hypertrophic parathyroid':ab,kw,ti OR 'parathyroid gland hypertrophy':ab,kw,ti OR 'parathyroid hyperfunction':ab,kw,ti OR 'parathyroid hypertrophy':ab,kw,ti OR parathyroid:ab,kw,ti OR hypoparathyroid*:ab,kw,ti OR 'hypo parathyroid*':ab,kw,ti OR 'hypoparathyreosis':ab,kw,ti OR 'hypoparathyroidy':ab,kw,ti OR 'parathyroid hypofunction':ab,kw,ti OR 'parathyroid insufficiency':ab,kw,ti OR parathormone:ab,kw,ti OR natpara:ab,kw,ti OR pth:ab,kw,ti OR 'parathorm':ab,kw,ti OR 'parathormon':ab,kw,ti OR 'parathormone':ab,kw,ti OR 'parathyrin':ab,kw,ti OR 'parathyroid hormone*':ab,kw,ti | 101,894 |
|  | 6. | 'hypercalcemia'/de OR 'hypocalcemia'/de OR 'hypocalciuria'/exp OR 'hypercalciuria' | 62,823 |
|  | 7. | hypercalcemi*:ab,kw,ti OR 'milk-alkali syndrome':ab,kw,ti OR hypercalcaemi*:ab,kw,ti OR hypercalciema:ab,kw,ti OR 'hypocalcaemi*':ab,kw,ti OR 'hypocalcemi*':ab,kw,ti OR 'hypocalcinemia':ab,kw,ti OR 'hypocalciuria':ab,kw,ti OR 'hypercalciuria':ab,kw,ti | 50,725 |
|  | 8. | 'calcium'/exp | 342,366 |
|  | 9. | ((calcium OR 'ca2+') NEAR/7 (increas* OR decreas* OR high* OR low* OR level* OR drop* OR down OR regulat* OR dysregulat* OR abnormal*)):ab,kw,ti | 167,251 |
|  | 10. | increas*:ab,kw,ti OR decreas*:ab,kw,ti OR high*:ab,kw,ti OR low*:ab,kw,ti OR level*:ab,kw,ti OR drop*:ab,kw,ti OR down:ab,kw,ti OR regulat*:ab,kw,ti OR dysregulat*:ab,kw,ti OR abnormal*:ab,kw,ti | 21,595,982 |
|  | 11. | 8 AND 10 | 233,905 |
|  | 12. | 4 OR 5 OR 6 OR 7 OR 9 OR 11 | 446,508 |
| Combined sets | | | |
|  | 13. | 3 AND 12 | 3,245 |
| Limits | | | |
|  | 14. | 3 AND 12 AND [1973-2020]/py AND [1973-2023]/py | 2,912 |
|  | 15. | 3 AND 12 AND [1973-2020]/py AND [1973-2023]/py AND ([danish]/lim OR [english]/lim OR [norwegian]/lim OR [swedish]/lim) | 2,713 |

Web of Science Core Collection (1975-present) 2023-03-09

Indexes=SCI-EXPANDED, SSCI, A&HCI, CPCI-S, CPCI-SSH, ESCI Timespan=All years

| Söktermer | | | Antal träffar |
| --- | --- | --- | --- |
| Lithium | | | |
|  | 1. | TS=("lithium*" or "lihionit" or "eskalith" or "lithotabs" or "lithane" or "dilithium" or "lithobid" or "lithonate" or "CP-15,467-61" or "CP15,46761" or "micalith" or "NSC-16895" or "NSC16895" or "priadel" or "Quilinorm-retard" or "Quilinormretard" or "camcolit" or "candamide" or "carbolit" or "carbolith" or "carbolitium" or "ceglution" or "contemnol" or "duralith" or "hypnorex" or "lentolith" or "licab" or "licarb" or "licarbium" or "lidin" or "limas" or "linthane" or "liskonium" or "liskonum" or "litheum" or "lithicarb" or "lithiumcarbonate" or "lithmax" or "lithobid" or "lithocap" or "lithocarb" or "lithionate" or "litilent" or "litocarb" or "maniprex" or "neurolepsin" or "phanate" or "plenur" or "quilonorm" or "teralithe" or "teralite" or "li-liquid" or "cibalith" or "litarex" or "mesin" or "lithium sulphate" or "Lithiophor" or "Lithiofor") | 288,892 |
| Hyper- or hypoparathyroidism, hyper- or hypocalcemia | | | |
|  | 2. | TS=("Hyperparathyroid*" or "hyper parathyroid*" or "hyperparathyroid crisis" or "hyperparathyroidea" or "hyperparathyroidosis" or "hypertrophic parathyroid" or "parathyroid gland hypertrophy" or "parathyroid hyperfunction" or "parathyroid hypertrophy" or "hypoparathyroid*" or "Hypo parathyroid*" or "hypoparathyreosis" or "hypoparathyroidy" or "parathyroid hypofunction" or "parathyroid insufficiency" or "Parathyroid" or "Parathormone" or "Parathyrin" or "Natpara" or "PTH" or "parathorm" or "parathormon" or "parathormone" or "parathyroid hormone*") | 83,197 |
|  | 3. | TS=("hypercalcemi*" or "milk-alkali syndrome" or "hypercalcaemi*" or "hypercalciema" or "Hypocalcemi*" or "hypocalcaemi*" or "hypocalcinemia" or "hypocalciuria" or "hypercalciuria") | 32,723 |
|  | 4. | TS=(("calcium" or "Ca2+") NEAR/6 ("increas*" or "decreas*" or "high*" or "low*" or "level*" or "drop*" or "down" or "regulat*" or "dysregulat*" or "abnormal*")) | 206,407 |
|  | 5. | 2 OR 3 OR 4 | 293,068 |
| Combined Sets | | | |
|  | 6. | 1 AND 5 | 1,141 |
| Limits* | | | |
|  | 7. | 1 AND 5 and English (Languages) | 1,092 |

- No articles were found in Swedish, Norwegian or Danish

PsycInfo via Ebsco 2023-03-09

| Söktermer | | | Antal träffar |
| --- | --- | --- | --- |
| Lithium | | | |
|  | 1. | DE "Lithium" OR DE "Lithium Carbonate" OR DE "Bromides" | 8,751 |
|  | 2. | TI ( lithium* or lihionit or eskalith or lithotabs or lithane or dilithium or lithobid or lithonate or "CP-15,467-61" or "CP15,46761" or micalith or "NSC-16895" or NSC16895 or priadel or "Quilinorm-retard" or Quilinormretard or camcolit or candamide or carbolit or carbolith or carbolitium or ceglution or contemnol or duralith or hypnorex or lentolith or licab or licarb or licarbium or lidin or limas or linthane or liskonium or liskonum or litheum or lithicarb or lithiumcarbonate or lithmax or lithobid or lithocap or lithocarb or lithionate or litilent or litocarb or maniprex or neurolepsin or phanate or plenur or quilonorm or teralithe or teralite or "li-liquid" or cibalith or litarex or mesin or "lithium sulphate" or Lithiophor or Lithiofor) OR AB ( lithium* or lihionit or eskalith or lithotabs or lithane or dilithium or lithobid or lithonate or "CP-15,467-61" or "CP15,46761" or micalith or "NSC-16895" or NSC16895 or priadel or "Quilinorm-retard" or Quilinormretard or camcolit or candamide or carbolit or carbolith or carbolitium or ceglution or contemnol or duralith or hypnorex or lentolith or licab or licarb or licarbium or lidin or limas or linthane or liskonium or liskonum or litheum or lithicarb or lithiumcarbonate or lithmax or lithobid or lithocap or lithocarb or lithionate or litilent or litocarb or maniprex or neurolepsin or phanate or plenur or quilonorm or teralithe or teralite or "li-liquid" or cibalith or litarex or mesin or "lithium sulphate" or Lithiophor or Lithiofor) | 11,936 |
|  | 3. | 1 OR 2 | 13,397 |
| Hyper- or hypoparathyroidism, hyper- or hypocalcemia | | | |
|  | 4. | DE "Parathyroid Disorders" OR DE "Parathyroid Hormone" | 269 |
|  | 5. | TI (Hyperparathyroid* or "hyper parathyroid*" or "hyperparathyroid crisis" or hyperparathyroidea or hyperparathyroidosis or "hypertrophic parathyroid" or "parathyroid gland hypertrophy" or "parathyroid hyperfunction" or "parathyroid hypertrophy" or hypoparathyroid* or "hypo parathyroid*" or hypoparathyreosis or hypoparathyroidy or parathyroid hypofunction or parathyroid insufficiency or Parathyroid or Parathormone or Parathyrin or Natpara or PTH or parathorm or parathormon or parathormone or "parathyroid hormone*") OR AB (Hyperparathyroid* or "hyper parathyroid*" or "hyperparathyroid crisis" or hyperparathyroidea or hyperparathyroidosis or "hypertrophic parathyroid" or "parathyroid gland hypertrophy" or "parathyroid hyperfunction" or "parathyroid hypertrophy" or hypoparathyroid* or "hypo parathyroid*" or hypoparathyreosis or hypoparathyroidy or parathyroid hypofunction or parathyroid insufficiency or Parathyroid or Parathormone or Parathyrin or Natpara or PTH or parathorm or parathormon or parathormone or "parathyroid hormone*") | 847 |
|  | 6. | (TI ( hypercalcemi* or "milk-alkali syndrome" or hypercalcaemi* or hypercalciema or Hypocalcemi* or hypocalcaemi* OR hypocalcinemia or hypocalciuria or hypercalciuria) OR AB ( hypercalcemi* or "milk-alkali syndrome" or hypercalcaemi* or hypercalciema or hypocalcaemi* OR hypocalcinemia OR hypocalcemi* or hypocalciuria or hypercalciuria )) | 306 |
|  | 7. | DE "Calcium" | 8,392 |
|  | 8. | TI ( (increas* or decreas* or high* or low* or level* or drop* or down or regulat* or dysregulat* or abnormal*) ) OR AB ( (increas* or decreas* or high* or low* or level* or drop* or down or regulat* or dysregulat* or abnormal*) ) | 2,661,041 |
|  | 9. | TI ( ((calcium or "Ca2+") N6 (increas* or decreas* or high* or low* or level* or drop* or down or regulat* or dysregulat* or abnormal*)) ) OR AB ( ((calcium or "Ca2+") N6 (increas* or decreas* or high* or low* or level* or drop* or down or regulat* or dysregulat* or abnormal*)) ) | 8,315 |
|  | 10. | 7 AND 8 | 6,816 |
|  | 11. | 4 OR 5 OR 6 OR 9 OR 10 | 12,220 |
| Combined Sets | | | |
|  | 12. | 3 AND 11 | 207 |
| Limits* | | | |
|  | 13. | 12 AND Limiters - Published Date: 19730101-20231231 | 200 |
|  | 14. | 13 AND Narrow by Language: - english | 189 |

- No articles were found in Swedish, Norwegian or Danish
